# Reflection on near-peer shadowing program: impact on operating room student’s perception of their future profession

**DOI:** 10.1186/s12909-022-03891-w

**Published:** 2022-12-01

**Authors:** Mahboobeh Khabaz Mafinejad, Fatemeh Ebrahimpour, Azadeh Sayarifard, Fatemeh Shahbazi, Larry Gruppen

**Affiliations:** 1 Health Professions Education Research Center, Education Development Center, Department of Medical Education, Tehran University of Medical Sciences, Tehran, Iran; 2 Department of Pediatric Nursing, Shahid Beheshti University of Medical Sciences, Tehran, Iran; 3 Center for Academic and Health Policy, Tehran University of Medical Sciences, Tehran, Iran; 4 Instructor of Medical Education, Department of Community Medicine, School of Medicine, Zahedan University of Medical Sciences, Zahedan-Dr. Hesabi Square-Medical Sciences Campus, Zahedan, Iran; 5 Education Development Center, Zahedan University of Medical Sciences, Zahedan, Iran; 6 PhD, Department of Learning Health Sciences, University of Michigan, Ann Arbor, MI USA

**Keywords:** Reflection, Operating Room Technicians, Students, Perception, Qualitative Research

## Abstract

**Background:**

Reflection is a key element in learning from observation and experience of future profession’s roles and responsibilities in clinical encounters. Moreover, reflection helps students cope with the challenges, complexities, and uncertainties of professional development. Students’ written reflections on clinical exposure offer valuable information, and their analysis provides instructors with invaluable insight into students' experiences. This study evaluated Operating Room students’ written reflections on their first clinical exposure experiences towards their future profession through the shadowing program.

**Methods:**

This study was a qualitative analysis on Operating Room freshmen’s reflections in the undergraduate program of Zahedan and Zabol University of Medical Sciences in Iran. After the shadowing program, all participants were asked to write an unstructured written reflection, and these fifty written reflections were de-identified and independently analyzed‏ using the thematic analysis approach.

**Results:**

Qualitative analysis extracted 10 subthemes and four main themes including (i) Moving towards the guiding realities of future profession, (ii) Discovering milestones of realizing professional identity, (iii) Managing the emotions affecting the perception of future profession’s desirability, and (iv) Excellence in professional growth and development.

**Conclusion:**

Reflecting on the experience of the shadowing program, the participants described being in the OR environment as a stimulating and valuable learning opportunity. Moreover, this experience helped improve their perception of future profession’s realities, as well as initiate realization of professional identity and planning for professional developments.

## Background

Transition from the preclinical to clinical phase is well recognized as a difficult and stressful period for many students [1–3]. Evidence indicated that many Operating Room (OR) students were not prepared for entering into the clinical environment in their preclinical phase. Windish et al. (2004) argued that educational programs were not sufficient to meet learners’ future needs in clinical settings [4]. Furthermore, most curricula do not provide OR students with opportunities to acquire practical skills, and thereby make them face challenges later in their clinical phase [5]. Challenges such as unfamiliarity with the clinical environment, clinical roles and responsibilities, high workload, as well as lack of self-confidence and adequate clinical knowledge and skills [6, 7] make such transition the most difficult stage of their education [8].

To facilitate the transition of students from preclinical to clinical phase, clinical exposure to workplace should be integrated from the early years of education [9, 10]. Studies showed that expansion of Early Clinical Exposures (ECE) for learners had a noticeable impact on preparing them for transition from academic environments to clinical settings, as well as enhanced their satisfaction, positive attitude towards future profession, interest and self-confidence [11, 12]. Thus, effective integration of the ECE program into medical education curricula like delivery of shadowing program was strongly suggested, and has been used by many educational institutions [13].

In the shadowing program, the learners are exposed to actual clinical situations to become familiar with professional responsibilities and processes, professional relationships, clinical workplace characteristics, and challenges [14]. Moreover, this program exposes the learners to the clinical environment, familiarizes them with responsibilities and roles, and helps them develop professional identity [15]. Evidence suggests that different educational institutions pursue various objectives through the shadowing program, including helping undergraduate applicants make an informed decision or helping graduate applicants choose a desirable specialty [16, 17]. Such programs provide active and participatory environments for learning through reflective observation of the teachers’ or seniors’ performance in actual clinical situations to enhance the learners’ perception of their future profession. As a way of modeling the importance of improving students' perception of their profession, near-peer shadowing programs can be an effective teaching modality for supporting professional development [18].

Evidence shows that reflection is a key element in learning from observation and experiencing their future profession’s roles and responsibilities in clinical encounters [19]. Reflection also helps students discover their needs throughout the course. Written reflection creates preconditions for students to analyze the experiences [20], and students’ written reflections on their clinical exposure offer valuable information [21]. The analysis of these reflections provide the instructors with invaluable insight into students' experiences [22]. However, previous studies have not typically used reflection to investigate OR students' perception on their first clinical exposures towards their future profession. They have also not examined how reflection can be used to influence such perception. The current study is unique in that (i) OR freshmen could shadow their near peers in the OR setting, and (ii) reflections, rather than survey data, were assessed. This study aimed to explore OR students' perceptions of near peer shadowing experience and investigating OR students’ reflections on their first clinical experiences towards their future profession through that program.

## Methods

This study was a qualitative analysis on Operating Room freshmen’s reflections in the undergraduate program of Zahedan and Zabol University of Medical Sciences in Iran. To understand the experiences of students in this program, we used qualitative analysis on students’ written reflections. Reflection is widely applied in qualitative studies [23, 24]. The academic literature on reflection has revealed that it can help us understand participants' internal dialogues and analyze their thought processes. On the other hand, written reflection is a well-documented data collection method, particularly in educational research [25].

### Setting

The traditional Flexnerian four-year undergraduate OR program’s curriculum in Iran is divided into preclinical (three semesters) and clinical phases (five semesters). The OR field of study is a branch of health sciences that teaches the principles of surgeries and new surgical technologies in specialized and sub-specialized surgeries, as well as care and help with patient management before, during and after a surgery. OR graduates become members of surgical teams to help perform surgeries in hospital ORs.

### Participants

The participants consisted of 50 OR freshmen; 27 (54%) female and 23 (45%) male from the undergraduate program at Zahedan and Zabol University of Medical Sciences in Iran. Students were newcomers to the undergraduate operating room curriculum. They had a mean age of 19.7 years (± 1.91).

### Procedure

In 2019–2020, during the paramedical pre-clinical curriculum, an 8-h shadowing program was implemented for OR freshmen as shadowees. Near-peer students in the clinical phase were considered as shadowers. Shadowees spent 10 h in two days with their assigned shadowers in OR setting. A shadowee made rounds with a shadower throughout multiple units in the OR to interact with staff members in the clinical setting. During the shadowing program, shadowees were invited to reflectively observe different settings of their future workplace, procedures, and events. Furthermore, in the near-peer shadowing program, shadowees could observe shadowers’ performance in everyday practice with no predefined agenda. Following the shadowing program, each shadowee had a period of reflection and debriefing with the assigned shadower.

### Writing a reflection

One research question regarded how shadowees describe and analyze their OR encounters. After the experience, before participants faced any other clinical experiences, shadowees were asked to write an unstructured written reflection on their near-peer shadowing experience. They reflected on their feelings, emotions, and thoughts during and following the program as well as lessons learned about their profession. The aim of the writing reflection was to describe notable events and interactions each shadowee may have observed during the program. The written reflections were mandatory requirement for participants. The reflections were not graded and did not mandate specific guidelines or writing style. This allowed shadowees to authentically and openly reflect upon their observations, experiences, and values most meaningful to them during the program. Fifty written reflections were collected from shadowees after the program.

### Data analysis

Written reflections were de-identified and independently analyzed‏ using thematic analysis by following Braun and Clarke’s (2006) approach [26]. Thematic analysis is used to gain insight into the experiences of OR freshmen in near-peer shadowing program. This approach helped explore perceptions into the phenomenon under study [27].

In this study, initially, all reflections were given an identification number. FSH and FEP studied all reflections and coded. After multiple readings of the reflection papers to familiarize the judges with all key contents and search for meaningful patterns, a list of ideas about what is interesting was generated and extracted. The meaning units were then carefully inspected, reflected upon, and assessed to detect new, more abstract aspects. The coding process was carried out by two authors (FSH and FEP) independently; raw inter-rater agreement was 95% (278/292). The cases of disagreement were discussed in a two-hour session with the participation of another expert (MKM), until consensus was reached. The emerging codes were organized into sub-themes and aggregated into main themes to explore participants’ perceptions. The themes were reviewed in terms of internal and external heterogeneity. If necessary, the themes were changed, and some new themes were introduced. An attempt was made to give the themes a short and concise name in such a way as to instill in the reader's mind what that theme is about. As the final stage of the analysis, we selected examples from quotes, finalized analysis of quotations, searched the literature, and prepared the scientific report.

To improve trustworthiness, Guba and Lincoln's criteria were used [28]. To ensure credibility, the researcher's previous acquaintance with the medical sciences education and its clinical and educational experiences was useful for understanding and analyzing the data. The codes, themes and subthemes extracted from the written reflection were checked with the participants as needed, and discrepancies were adjusted. For confirmation ability, two members of the research team analyzed the data independently and reached a consensus in the session. To ensure dependability, the researchers conducted semi structured questions using a written reflection guide to explore the topic of interest. Transferability was achieved by describing the research context thoroughly and providing detailed explanations of the research process.

## Results

Overall, while OR freshman students had been exposed to the near-peer shadowing program, didactically and clinically, many noted that these experiences helped them to reflect on the profession, and transition from preclinical to clinical stage. Findings showed that 10 subthemes and four main themes including (i) Moving towards the guiding realities of future profession; (ii) Discovering milestones of realizing professional identity; (iii) Managing the emotions affecting the perception of future profession’s desirability and (iv) Excellence in professional growth and development. Main themes, sub-themes and examples of participants' quotes are presented in Table 1.

**Table 1:** Main themes, subthemes, codes, and examples of participants' quotes

Theme	Sub-theme	Example Quote
**Moving towards the guiding realities of the profession**	Emotional atmosphere	"I did not have a positive impression of the OR environment until I entered it. A dull, dry, and cold environment. But, when I saw the OR, I realized that it was not as I thought.” (No.3-female (
	Environment and setting	"Before this experience, I considered the OR to be a place where things are difficult to do. But, after this experience, I realized that if we look at it in a positive light, we can appreciate working in it." (No.12-male)
**Discovery of milestones forming a professional identity**	Understanding the realities of OR-related professions’ nature	“Before entering the OR, I thought my job was simple and repetitive, and my role didn't matter so much. So, I didn't feel good. But, after visiting the OR, I enjoyed helping others and being useful in the OR.” (No.15-female)
	Recognizing expected professional competencies	“The OR discipline requires precision and practical skills. This discipline is interesting and at the same time sensitive” (No.2-female)
**Managing emotional reactions affecting the perception of OR discipline’ desirability**	Positive feeling and emotion about future profession	“The doctor came and started the surgery. I was very excited to watch it up close and started asking questions from seniors.” (No.6-female)
		“I am very happy with this experience, and I was very excited when I entered the OR.” (No.1-male)
	Satisfaction with choosing OR profession	“I'm glad I chose an exciting and interesting field of study.” (No.4-female)
		“Now that I rethink about it, I see that the OR is a very good discipline, well-suited with my personality type. It suits my morals and emotional state. These make me happy with my choice.” (No.11- female)
	Psychological adjustment to personal emotions	“The first surgery that I observed was very scary. I felt I could not face the blood. My hands were cold, but I was trying to comfort myself.” (No.16- male)
**Excellence in the path of professional development and growth**	Motivation for professional excellence	“I dreamed of it before being accepted in this field of study, and now after visiting the OR and observing surgeries, my interest in my field increased.” (No.19- female)
		“This experience introduced me to my future profession, which increased my motivation to continue this field of study.” (No.13- male)
	Goal setting for professional growth and development	“I decided to set a goal to become a good and successful OR technologist.” (No.8- female)
		“I have to try to save life of others and help the sick as much as I can.” (No.4-female)

### Theme I: moving towards the guiding realities of the profession

This theme consisted of familiarity with facts and realities that led to a change in participants' perceptions of their future working conditions and profession. Participants' perception of the emotional atmosphere and environment shows that OR freshman students had misconceptions or inadequate information about the profession and the clinical environment. The near–peer shadowing program led to more accurate and realistic perceptions of interaction with the environment. Most students perceived the clinical environment as a negative emotional and physical atmosphere that was non-empathetic, hard, and stressful. They were able to experience a more positive clinical environment after experiencing the near–peer shadowing program. They achieved a realistic understanding of the OR environment and the profession. They found the clinical environment as an enjoyable learning environment with good teamwork and examples of an individual's ability to manage stress.

Participating in the shadowing program resulted in changing perspectives on inappropriate professional interactions of treatment team members. There were also changes in understanding effective inter-professional communication as well as correcting perceptions on communication with patients in OR environment. A participant mentioned:

"I did not have any OR experience before this program. My impression of OR, according to those around me was that no attention was paid to the patient, and the treatment team and doctors did not sympathize with the patient. When I entered the OR, I saw a good atmosphere between the patient and the nurses." (Student No.1, Female).

In addition, another student stated:

"Before being in OR environment, I had a different perception and I had a strange fear and anxiety. But, contrary to my expectations, the OR had a good atmosphere and my fear decreased to some extent." (Student No.12, Female).

Before encountering the OR, most students described their perception of the OR as an unfriendly, insensitive, gloomy, and difficult working environment. Some students described their mental image of the OR environment as a sophisticated and modern environment. After participating in the program, OR environment was described by most students as a stimulating and dynamic. A student mentioned:

"Before this experience, I considered OR to be a place where things are difficult to do. But, after this experience, I realized that by looking at it in a positive light, we can appreciate working in it." (Student No.17, female).

### Theme II: discovering milestones of realizing professional identity

Sub-themes related to “Discovering milestones of realizing professional identity” were understanding the facts about the nature of the profession and recognizing expected professional competencies. Understanding the nature of the profession, as well as the expected competencies as a result of participating in the shadowing program, helped students develop a professional identity from the first days of entering this field of study. Understanding the OR-related profession’s vital roles, recognizing various and multi-faceted tasks, as well as experiencing its usefulness in providing services to the patient, helped students realistically comprehend future profession’s nature. Most participants stated that the shadowing program provided the opportunity to reflect on their professional roles, tasks, and duties.

"While I realized my tasks were difficult, I became more interested in my field of study." (Student No.16, female).

"Before entering the OR, I thought I had an easy job. But, during this program, I became familiar with the working environment and various tasks I have. I learned how to do things and treat others. Also, I learned about my role in OR, my responsibilities, as well as OR team members, my communication and interaction with them. I also learned about the value of my field of study." (Student No.3, male).

The opportunity to get acquainted with the various expected competencies in the profession by participating in the program was frequently mentioned by most students in the written reflections. Adherence to professional behaviors, effective communication skills with patients, colleagues, and other treatment team members, acquiring clinical skills and personal development for self-directed learning were among competencies for working in OR. About learning communication skills by observing shadowers’ performance, a student stated:

"I learned that OR requires strong collaboration among people on the surgical team. The interactions among the team were very interesting to me." (Student No.4, male).

Another participant stated:

"I need to improve, with more effort, my skills and knowledge in both theory and practice so that I can perform better in the OR and take care of health of my fellow human beings." (Student No.23, female).

### Theme III: managing the emotions affecting the perception of future profession’s desirability

Managing professional emotions and feelings, creating a sense of satisfaction with the profession and effective psychological adjustment to personal emotions, make the profession desirable and acceptable. Once students enter the undergraduate program in their chosen field of study, they experience negative and sometimes contradictory emotions during their educational program due to lack of exposure to the related work environment and consequently lack of effective knowledge of the profession. According to the participants, encountering the OR environment during the shadowing program stimulated positive professional emotions and effective management of emotions in clinical settings, especially the OR environment. Moreover, direct and close observation and experience of OR environment and surgeries stimulated feelings of enthusiasm and curiosity in students to learn more. A student stated:

“The day the program coordinator announced that we were going to take the first-semester freshmen to become familiar with OR, I had a very strange feeling. I never thought I could see the OR so soon. From the moment I heard the news, I was expecting the day, and finally, the promised day arrived. From the moment I put on gowns, my eagerness increased. What is more important than getting acquainted with your future job environment? Seniors told us our passion clearly showed that it was out first time in OR." (Student No.14, female).

As a shadowing program’s outcome, several students referred to psychological and emotional adjustment (e.g., managing fear and anxiety) after learning about the OR environment and observing OR team’s collaboration and communication. In addition, several students acknowledged the noticeable role of shadowers in managing freshmen’s fear and anxiety and supporting effective learning in the OR environment. A participant said:

"With the start of the surgery, my stress and anxiety decreased because of the encouragement due to good cooperation and close relationship of the OR staff." (Student No-14, male).

According to the participants, as a result of ensuring that the future profession fits personal characteristics and expected goals, they became realistically satisfied with their chosen field of study. A student mentioned:

"Now, I think OR is a very good field and it fits my personality, morals and emotions, and that makes me happy with my choice." (Student No.3, male).

### Theme IV: excellence in the path of professional growth and development

Participating in a shadowing program helped students achieve a positive and productive motivation for professional excellence and plan for further career growth. The students emphasized their strong intention to continue their studies at higher levels as well as to acquire and strengthen their professional qualifications. As a key principle, moving towards excellence in the profession requires achieving the highest professional performance and ultimately serving the community. A student reflected:

"I intend to improve my skills and knowledge so that I can perform better in OR and make every effort for improving human health " (Student No. 23, male).

Also, another participant pointed out that participating in this program provides a great opportunity to rethink career goals to achieve the future professional aspirations. One participant stated:

“My plan for the future is to become one of the skillful OR technicians in my country.” (No.2-male)

## Discussion

Operating Room freshmen joined seniors in the OR environment in a shadowing program, and then wrote reflection papers about their first clinical experience. According to the qualitative analysis of written reflection, participants gained a genuine experience of professional realities, understood future profession’s desirability, laid foundations to form professional identity and to plan for future profession’s growth and excellence.

Analysis of the reflection papers indicated that the shadowing program helped initiate the formation of a professional identity by providing rich learning opportunities that promoted students’ understanding of the importance of acquiring professional competencies and future professional identity. Participants believed that associating with peer seniors improved their understanding of the profession’s core competencies. Moreover, through their encounter with the clinical environment, participants became acquainted with their future profession’s realities early in their studies. By providing situational learning opportunities, the shadowing program helped freshmen gain a realistic understanding of professional competencies through interacting with others. The present findings were supported by those of other studies in this field [29–31].

The present study shows that participating in this program led students to attend to various issues such as responsibility, effective collaboration among team members, and effective communication and interaction with patients and other healthcare team members. This program also provided participants with the opportunity to reflect on and improve communication skills and interprofessional collaboration. Given the sensitivities of working in the OR environment, collaboration among healthcare team members is vitally required. Similar studies highlighted the effects of implementing such programs on interprofessional interactions [32, 33]. It seems that due to the culture of academic competition among students in underdeveloped countries, communication and interaction skills are often neglected [34]. According to the results of our study, the implementation of such programs can lead to a better understanding of the importance of improving communication and teamwork skills among students. Fougner et al*.* (2011) stated that students were able to think about their future role in inter-professional team communications as a result of being in a clinical setting [35]. Von der Lancken et al. (2018) used a shadowing program to help clinical graduates become acquainted with other professions. Its implementation raised awareness of interprofessional relationships, increased knowledge regarding roles and their identity, and enhanced beliefs regarding interprofessional collaboration [36]. Wright et al*.* (2012) highlighted that students, in their own reflections, noted the importance of communication skills with patients. The way of communication among healthcare team members was interesting for them and they clearly observed the effects and outcomes of effective communication in the patient's treatment process [33].

Participants in this study also learned about the nature of their profession through understanding of the profession’s life-saving role, multiple multi-faceted roles and tasks, and position in the OR team. Kusnoor et al. (2016) showed that the shadowing program provided an opportunity for students to become familiar with the expected OR’s roles and responsibilities and even other disciplines [37]. Monahan et al*.* (2018) also stated that the implementation of such programs provided students with a better understanding of the future profession’s roles and responsibilities [30]. The present findings suggest that the knowledge gained about roles and responsibilities in a team through observation in a clinical setting provided many examples of real-world situations, which can be used by students in future.

The OR students, in their reflections, pointed out that this program was effective in revising their beliefs about the OR’s prevailing atmosphere and improving their understanding of OR’s emotional situations, physical environment, and facilities [38]. In various studies, the OR environment was mentioned as a stressful and traumatic environment for students [38] that its prevailing atmosphere could affect students' learning [39]. According to the present findings, implementation of shadowing programs, as their first clinical exposure experience, could help change students' perceptions of OR’s prevailing atmosphere.

According to the present study, students achieved a broader view of OR equipment and tools, and their functionality. After this program, students were closely acquainted with the tools and equipment and learned their application accurately and in practice in the real OR’s working environment introduced in theoretical lessons in form of simulations or illustrations. Through such programs, students can well establish a bridge between theoretical and practical courses and observe the application of theory in practice. Similar studies were conducted to fill the gap between clinical settings and classrooms [40]. In other words, providing the conditions for students to enter the clinical setting before their clerkship necessary, and this program enabled students to understand the application of learned material in practice. It causes to prepare students for performing as a the doctors of tomorrow [41]. The present findings are in line with those of other studies that implemented such programs for familiarizing pre-clinical students [42].

According to analysis of students' reflection, excellence in professional development and growth occurred through arousing interest, motivation, and goal setting for performance advancement as a result of participating in the shadowing program. Moreover, the shadowing program enhanced their interest and motivation not only to work in their chosen profession, but also to be on the path of professional growth and excellence. Additionally, they become familiar with their future roles and responsibilities as a surgical technologist, they became motivated to plan carefully for their progress and success, and they were motivated to ponder about opportunities for future professional accomplishments.

As a main theme of the present study, tackling emotional reactions was observed to affect the students’ perception of the desirability of their chosen field of study, i.e., when freshmen entered the OR, they experienced being pleased about choosing their field of study. Analysis of participants’ reflection papers revealed that by getting acquainted with the facts and realities of their chosen field, their previous vague mental image of the OR environment was revised, and deeper knowledge of their profession enhanced their motivation and interest towards their future profession [43].

The present study has some limitations including the exposure experience in the OR environment; hence the present findings may not be generalizable to other clinical educational settings. Moreover, using the unstructured reflection can help participants freely express views and experiences on their personal experience. Although participants were initially informed that in exchange for writing reflections, no recompense or incentive was offered, they might still have answered reflection questions such that the answers please the researchers. As an advantage of this study, the reflection papers were written by students after being in OR environment and analyzed immediately; this approach can help educational planners for integrating it on educational opportunities and can lay foundations for further studies.

## Conclusion

This study investigated OR students' perception on their first clinical exposures towards their future profession using reflections, rather than survey data. Unstructured reflection can encourage participants to freely express personal views and experiences. Participants described this program as a pleasant and valuable learning opportunity that helped them to improve their understanding of future profession’s realities as well as initiate professional identity formation and planning for professional growth. The findings showed that when students enter the clinical setting, they pay attention not only to the subjects taught in the preclinical phase but also to such issues as how to interact with and respect patient's rights. Although they may not have targeted training in the shadowing program, it made the students implicitly aware of the need to improve the expected competencies in the field of communication skills and professionalism from the initial days of the curriculum. According to the results of this study, the implementation of the shadowing program has great potential to help the professional growth of OR students in their preclinical phase.

## Data Availability

The datasets generated and/or analyzed during the current study are not publicly available due the data are in Persian language but are available from the corresponding author on reasonable request.

## Abbreviation

OR: Operating Room
